# Conceptual framework of equity-focused implementation research for health programs (EquIR)

**DOI:** 10.1186/s12939-019-0984-4

**Published:** 2019-05-31

**Authors:** J. Eslava-Schmalbach, N. Garzón-Orjuela, V. Elias, L. Reveiz, N. Tran, E. V. Langlois

**Affiliations:** 1 Hospital Universitario Nacional de Colombia, Bogotá, Colombia; 2 Clinical Research Institute, School of Medicine, Universidad Nacional de Colombia, Bogotá, Colombia; 3 Evidence and Intelligence for Action in Health Department Pan American Health Organization, Washington, USA; 4 World Health Organization, Geneva, Switzerland; 5 Alliance for Health Policy and Systems Research, World Health Organization, Geneva, Switzerland

**Keywords:** Health equity, Health programs, Implementation research

## Abstract

**Background:**

Implementation research is increasingly used to identify common implementation problems and key barriers and facilitators influencing efficient access to health interventions.

**Objective:**

To develop and propose an equity-based framework for Implementation Research (EquIR) of health programs, policies and systems.

**Methods:**

A systematic search of models and conceptual frameworks involving equity in the implementation of health programs, policies and systems was conducted in Medline (PubMed), Embase, LILACS, Scopus and grey literature. Key characteristics of models and conceptual frameworks were summarized. We identified key aspects of equity in the context of seven Latin American countries-focused health programs We gathered information related to the awareness of inequalities in health policy, systems and programs, the potential negative impact of increasing inequalities in disadvantaged populations, and the strategies used to reduce them.

**Results:**

A conceptual framework of EquIR was developed. It includes elements of equity-focused implementation research, but it also links the population health status before and after the implementation, including relevant aspects of health equity before, during and after the implementation. Additionally, health sectors were included, linked with social determinants of health through the “health in all policies” proposal affecting universal health and the potential impact of the public health and public policies.

**Conclusion:**

EquIR is a conceptual framework that is proposed for use by decision makers and researchers during the implementation of programs, policies or health interventions, with a focus on equity, which aims to reduce or prevent the increase of existing inequalities during implementation.

## Background

### What is implementation science and implementation research?

Implementation is “*the process of putting to use or integrating new practices within a setting*” [1] and includes within its considerations relevant aspects such as the communities where it is thought to be carried out, the barriers and facilitators for it, the specific needs of the population, which differ for each intervention, for each type of country and region within each country [2–5]. This makes it very difficult to study it scientifically, which has motivated the progress of Implementation Science (IS) [3, 6], which is defined as “*the systematic study of how to design and evaluate a set of activities to facilitate successful uptake of an evidence-based health intervention*” [7].

In addition, the implementation is different for preventive and curative services, which in many countries are carried out by different funding sources and participants, which makes the implementation and systematic evaluation of it even more complex [8]. The attitude of the population also varies, which generates inequalities in health and in general, because of the different levels of the social determinants (education, occupation, place of residence, socioeconomical status, race / ethnicity, etc.) [9–12] which also impact the results of any type of implementation that does not consider or cannot act on the determinants. As a result of all the above, the need arises to make a systematic evaluation of the implementation using the tools of the IS.

Implementation Research (IR) covers the systematic use of the scientific method for the IS and can be defined as research that “*identifies common implementation problems and main determinants which hinder effective access to interventions; develops and tests practical solutions to these problems that are either specific to particular health systems and environments, or that address a problem common to several countries in a region; and determines the best way of introducing these practical solutions into the health system and facilitates their full scale implementation, evaluation and modification as required*” [13]. IR is, in other words, a scientific approach for implementing and assessing implementation of health policies, programs or interventions on hierarchy-embedded implementation outcomes, ranging from process outcomes, through implementation outcomes, to population health outcomes [14].

### Why is IR research different in general?

Even when IR uses the available tools of the scientific method, its objective of study is the implementation of health policies, programs or interventions, which makes it different from classical research that focuses on finding the effect of such policies, programs or interventions, without considering all the aspects that affect this effect during implementation [14]. With this method, IR evaluates the effect of such policies, programs or interventions in the community after implementation, finding scientific evidence on the real impact of implementation, based on short, medium or long-term indicators [13, 14]. It is evident that there is still a gap in the implementation of highly effective strategies in controlled studies, which fail to demonstrate such effectiveness after their implementation [15–17], and this gap is even greater in the evaluation of the impact of these interventions on the increase or decrease of existing inequalities during implementation. IR offers the possibility of evaluating this effect during implementation, and in the case of this proposal, equity-focused IR offers the possibility of intervening and evaluating the effect on equity with the IR [11, 18].

Several frameworks have been used in implementation sciences, including the Quality Implementation Framework [19], the Consolidated Framework for Implementation Research (CFIR) [15] and Promoting Action on Research Implementation in Health Services (PARISH) [20]. The CFIR was proposed to be used for evaluating impact of health equity research during the phase of exploring the underlying mechanism of disparities and during the phase of developing and evaluating interventions to reduce disparities [15, 21]. However, these IR frameworks do not include explicit health equity considerations during the whole implementation process, and do not help determine whether the implementation could positively or negatively affect avoidable and unjust inequalities in health [22]. At the time Braveman had proposed a conceptual framework for monitoring equity in health and healthcare, with 8 steps to follow, in which the last step was responsible for developing a strategic plan for implementation, monitoring and research, taking into account the political and technical obstacles, based on inequities or inequalities previously found, but without explicitly including the steps to be included during the implementation to improve or not increase said inequities [23]. The focus of the monitoring was based more on the documentation and monitoring of inequalities than on implementation [23].

Since 2014, the Alliance for Health Policy and Systems Research (AHPSR), an international partnership hosted by the World Health Organization (WHO), in collaboration with the Pan-American Health Organization (PAHO), has worked to facilitate the implementation process of programs, policies or health interventions, using evaluation research tools embedded in the implementation process [24]. This process focuses on embedding research within existing processes in order to shine light on context-specific factors related to real world health program, policy and system decisions – including implementation of health interventions - identified by people working within health systems. Implementation research is, in this case, an approach that could diminish the negative impact of implementing new interventions on health inequalities (differences in health among individuals or groups) or health inequities (differences in health, that are avoidable, unjust and unneeded) [9, 25]; or it could even be used to diminish existing inequalities or inequities identified in a population, with the implementation of new technologies, for instance.

To guide future research and practice, there is a need to develop an equity-based framework for implementation research of health programs, polices and systems that could be used to improve evidence-informed implementation processes. The aim of this study is to develop and propose an equity-based framework for Implementation Research (EquIR) of health programs, policies and systems.

## Methods

This proposal was developed in three phases:

1. We conducted a systematic review of the literature (published previously [26]) to identify conceptual frameworks or models that incorporate aspects of health equity into implementation research in Medline (PubMed), Embase, LILACS (1965–2016), and Scopus (1998–2016) databases and grey literature. The search strategy was composed of words related to “implementation” (implement* OR operations OR delive* OR implementation science OR (translational AND (science OR research OR Medical Research)) OR quality improvement OR task shifting OR policy OR Implementation Research) and Equity in health (Health equity OR health inequ* OR health disparit* OR vulnerable population OR advantaged population OR disadvantaged population). The search was not limited by language and there were no exclusion criteria. All related titles were included after eliminating duplicates. Three independent reviewers rated the non-relevant articles and categorized the articles.if they met the following criteria: implementation research, the science of implementation, and health equity. The data extraction form included also categories of health equity, implementation research, and kind of models or frameworks. We did not evaluate the quality of articles describing models and frameworks, as these were descriptive reports. Further details of the methodology for this systematic review have been published elsewhere [26].

2. We conducted a stakeholder’s analysis based on the work conducted by the Pan American Health Organization and the Alliance for Health Policy and Systems Research). Since 2014, the two organizations have developed a country-focused program to facilitate improvements in program, policy and system implementation through research embedded within existing processes.

The stakeholder analysis involved decision-makers and researchers of seven health programs that were receiving funding and support for conducting an implementation research study, during 2016–17. These stakeholders were selected following a call for the improving Program Implementation through Embedded Research (iPIER), throughout all Latin American countries, where aspects related to the disadvantaged population were included in the application. The winners of that call were those who formed part of this process and were made up of researchers and decision makers (local policy makers) of the region where the program implementation would be made. Henceforth we will call them “implementers”. We gathered information related with to the awareness of inequalities in health policy, systems and programs, the potential negative impact of increasing inequalities in disadvantaged populations, and the strategies used to reduce them. For example, implementers were asked key questions related to health equity such as: “Who is your health program targeted at?” and “during program implementation, have you monitored the effect of any disadvantaged group or others population?”. This was aimed at identifying the inclusion of equity issues in implementation research endeavors.

During a follow-up period, implementers were accompanied by a group of mentors who helped with the work of conducting the research under the framework of implementation research, selecting options to improve the program, or policy and planning its implementation. Protocols were critically reviewed under the equity perspective, and a framework proposal was discussed in-person with each one of the final seven groups of implementers during a workshop meeting. Lessons learned during the whole process were used to improve the framework according to the experience and knowledge of the implementers. The suggestions mentioned by the previous groups were included in the global analysis by the following groups for their consideration, and after they were ratified, they were included in the definitive framework. All participants are included in the acknowledgments section.

3. Finally, the implementation research group, and health equity experts used the findings from the systematic review and the experience of the country teams to build a framework called the Equity-Based Framework for Implementation Research (EquIR). Face-validity of the framework was assessed by key experts in the field, including through interviews with stakeholders from AHSPR, and PAHO, as well as decision-makers and researchers involved in the health programs, during the workshops, as mentioned in Phase 2. This framework is intended to be used to support the application of an equity-lens to implementation research proposals, and to facilitate the implementation of equity-focused health interventions and programs. We provided a practical example applied across EquiIR steps, using the “Mi Salud” program implemented in Bolivia within Phase 2.

## Results

### First phase

The systematic review of models and approaches involving equity in the implementation of health programs found 19 articles: 12 of them were general models, 5 included topics related to ethnic/racial disparities, and 2 were related to children’s health. Additional issues mentioned in the models included: funding, infrastructure, governance, quality, internal barriers and coverage [26]. Although there was no consolidated model to explicitly include equity issues in implementation research [26], the models included essential characteristics that were further incorporated into our framework. Table 1 shows some of the equity issues mentioned separately in those models, including planning, monitoring, designing, implementing and identifying disadvantaged population; these are the main topics considered as part of the development of the framework proposed in this paper.

**Table 1:** Relevant issues used for the development of the conceptual framework of Equity-focused Implementation Research for Health Programs (EquIR)

Models	Aspects of equity	Relevant issues for the development of the EquIR conceptual framework
National framework for health sector monitoring, evaluation, and analysis [27]	General	• Monitoring and evaluation if implementation: access and availability of services, coverage of interventions and impact (health condition, ability to respond)
Impact evaluation framework [28]	General	• Program Planning: Effectiveness analysis, equity analysis, health systems analysis, scale-up analysis, and policy analysis
Framework for strengthening health systems [29]	General	• Program Planning: Benefits for strengthening health systems
Promoting Action on Research Implementation in Health Services (PARISH) [20]	Race/Ethnic	• Program Planning: A diagnostic and evaluative measure of evidence and context elements. • Design: Determination of the most appropriate facilitation method.
Child Health and Nutrition Research Initiative (CHNRI) [30]	Children’s health	• Program Planning: o Research question: description, delivery, development and discovery research. o Identification of disadvantaged group: prioritization of research ideas in terms of answerability, effectiveness, deliverability, maximum potential for disease burden reduction, and effect on equity • Design: facilitated consensus development through measuring collective optimism.
Conceptual Model for Racial and Ethnic Disparities in Healthcare [31]	Race/Ethnic	• Program Planning: make recommendations for future interventions to reduce disparities
Implementing health promotion tools in Australian Indigenous primary healthcare [32]	Race/Ethnic	• Program Planning: Participation agreements, orientations, and training. • Design: Quality assessments, feedback and action planning. • Implementation tools
Large-scale fortification of condiments and seasonings as a public health strategy: equity considerations for implementation [33]	General	• Implementation of equity strategies: Enhancing the capabilities of the public sector, improving the performance of implementing agencies, strengthening the capabilities and performance of frontline workers, empowering communities and individuals, and supporting multiple stakeholders engaged in improving health.
Equity-focused knowledge translation toolkit [34]	General	• Getting ready, starting in the right place and developing a comprehensive strategy. • Building a coalition of partners, determining the current challenge (planning your equity-focused knowledge translation strategy), and clarifying your intended audience

Source: Authors, adapted from Eslava-Schmalbach J, Garzón-Orjuela N, Elias V, Reveiz L. Equity Incorporation in health in the Implementation Research: a review of conceptual frameworks. Rev. Panam Salud Publica. 2017;41:e126. doi: 10.26633/RPSP.2017.126 [26]

### Second phase

A summary of the projects involved in the implementation research program is shown in Table 2. Initially, all 7 projects included disadvantaged population in their proposals. However, in the course of developing an implementation research project, only two groups maintained an equity focus throughout the entire project and maintained this focus in the development of the research protocol.

**Table 2:** Equity issues in the implementation research proposals

Country		IR theme	Equity consideration	Disadvantaged Population
Argentina	Before	“Health policies implementation unit for the imprisoned population in Buenos Aires.”	To evaluate the possibility to implement focused strategies for disadvantaged populations inside the prisons.	Authors identified transgender individuals as more disadvantaged population inside prisons and developed a focused strategy for them. However, it was not explicit in the initial proposal
	After	“Barriers and facilitators of a tuberculosis prevention and control implementation program in imprisoned population, in Buenos Aires”		
Bolivia	Before	To identify barriers to “Nutritional Chispitas” in children 6 to 23 months old attending a primary healthcare center in the Andean Health Network	Children that do not attend these programs present greater social disadvantages	Children 6 to 23 months old of the Andean Health Network including those who go and who do not go to the primary healthcare centers and receive care under “Mi Salud” Program
	After	To identify barriers and facilitators to “Nutritional Chispitas” in children 6 to 23 months old attending primary healthcare centers, and by Mi Salud Program		
Brazil	Before	Psychosocial Attention Network Qualification Program (RAPS)	There is no mention of a socio-economic disadvantaged population with higher risks of mental illness	It was not included in the final version of the proposal. It was suggested to consider a population with mental illness, specifically those with a higher grade of social disadvantage. It was suggested to include them in the analysis.
	After	Implementation research of strategies to strengthen leadership to guarantee the rights in the CAPS of São Bernardo do Campo / SP.		
Chile	Before	Policy on interchangeability of medications in Chile	It was suggested to the authors to evaluate the impact on out-of-pocket payments related to medicines	The authors focused the project on the private market. However, it won’t be possible to know the impact on different income quintiles of the population, because this information is not available in the database.
	After	Medication interchangeability policy implementation of medicaments in Chile		
Colombia	Before	“Por ti Mujer” Program for early detection and treatment of women with cervical anomalies.	The program could implement strategies of vertical equity to improve the adherence of the more disadvantaged population.	A disadvantaged population is not identified in the final version. However, it was suggested to the authors to analyze the population from the perspective of ethnicity and a socio-economic variable.
	After	“Por ti Mujer” Program		
Perú	Before	Inter-programmatic articulation of tuberculosis and mental health for the National Tuberculosis Prevention and Control health strategy	Adherence to TB treatments of patients with mental disorders is a problem. However, it was suggested to consider also TB patients with mental disorders that are not near healthcare centers, and can be further disadvantaged.	The initial proposal and researchers were changed. Callao is a Peruvian region with a high social disadvantage in many aspects -economic, access to healthcare, standard of living, population density, sanitation, etc. A multi-sectoral approach was suggested considering these social determinants of health.
	After	Implementation factors and treatment adherence of the Tuberculosis Prevention and Control National health strategy at Callao - Perú		
Dominican Republic	Before	Family planning program	The perspective of gender equity is included from the beginning. It was suggested to consider male adolescents that do not have access to the family planning program, given that they are usually in a more disadvantaged condition than those who really do.	The socio-economic perspective is not identified in this new version. However, it was suggested to include it in the analysis.
	After	Gender and contraception in the Dominican Republic: a look at men		

Source: Authors

In a meeting with the country teams, the inclusion of equity-issues in the analysis phase of the projects was suggested to all participants. The absence of a conceptual model to guide the inclusion of equity issues during implementation research of health programs was evident. A draft version of the conceptual model was discussed during this meeting, and some of their suggestions were included in the final version of this proposal.

### Third phase

**Fig. 1:**
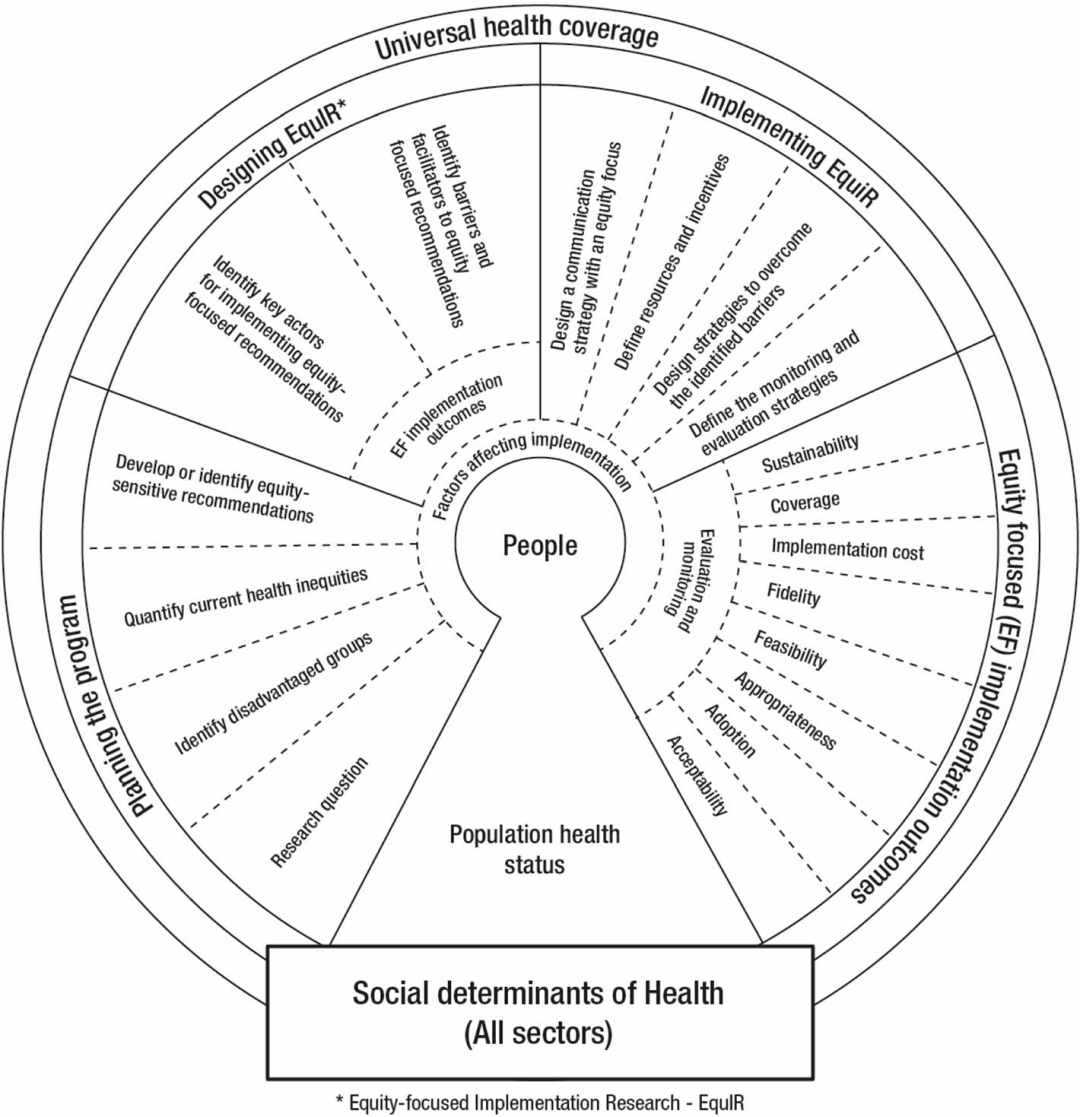
Conceptual framework of Equity-focused Implementation Research – EquIR. Source: Authors

**Fig. 2:**
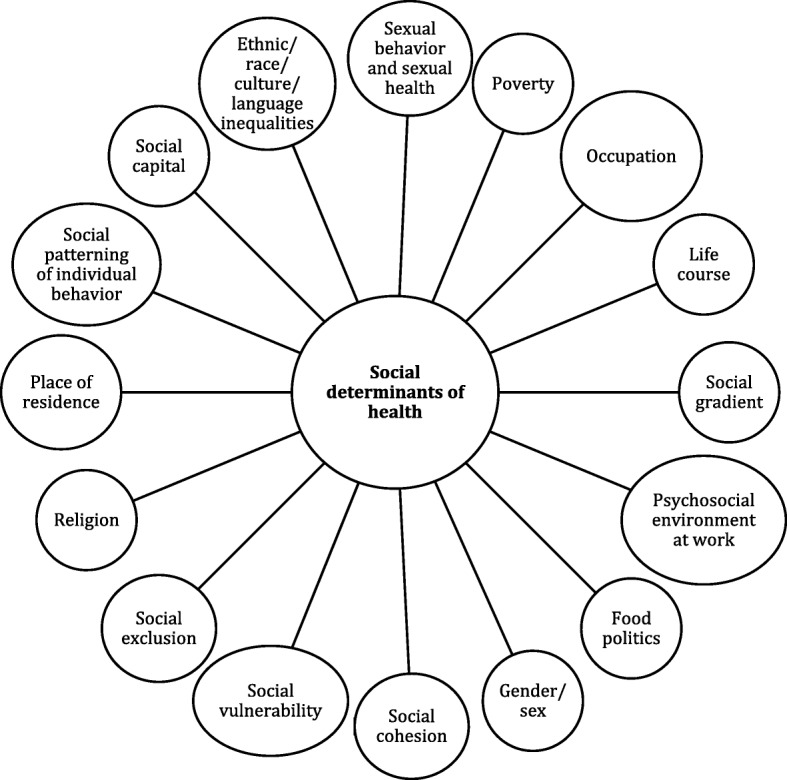
Social determinants of health. Source: Authors, adapted from Marmot M. Social determinants of health inequalities. Lancet. 2005 Mar;365(9464):1099–104. DOI: 10.1016/S0140-6736(05)71146-6

Finally, a conceptual framework of Equity-focused Implementation Research for Health Programs (EquIR) was developed (Fig. 1). It includes elements of Implementation Research beginning with a previous equity-focused population health status and finishing with a new population equity-focused health status. This is an iterative process that could be repeated until the IR outcomes and/or equity-focused health status of the population is actually improved. Additionally, other sectors (work, agriculture, health, economic, technology and innovation, education, social welfare, environment, culture, transport and others) were included in association with the social determinants of health [35], universal health coverage and the potential impact of the EquIR of health programs. This is what is called the context and could be related directly to the implementation within the health system/sector, or indirectly, by other sectors. Social determinants of health are highly relevant in the occurrence of health problems and disparities related to health problems (Fig. 2) [36]. They could be operationalized with the use of the PROGRESS Plus proposal (the elements of “PROGRESS” are Place of residence, Race/ethnicity/culture/language, Occupation, Gender/sex, Religion, Education, Socioeconomic status and Social capital, and “Plus” captures other aspects of discrimination and health disadvantage, like age, disability, sexual orientation and transitions) [37, 38].

Clockwise, the starting point of the conceptual framework of Equity-focused Implementation Research – EquIR (Fig. 1) is a population’s health status. The suggested steps in this framework are:

- 1st Step: To identify the health status of the population as the starting point in each cycle and as the focus of the health program or intervention. This step must include the health status of the general population as well as of the disadvantaged population. It is a crucial step because it could affect the results of the program, and specifically, the results for the disadvantaged population. In the case of Bolivia, the program was going to be implemented in a disadvantaged community of children. However, at the beginning it focused on children with the possibility of access to a primary healthcare center. Within this community, children unable to attend a healthcare center are more disadvantaged than others and would not benefit from the program. Consequently, the authors changed the way of implementing the program with a view of having a positive effect on children living far away from health centers.
- 2nd Step: In the equity focused planning phase of the program (EquIR Planning Phase), it is important to identify the relevant research questions, taking into consideration the disadvantaged populations potentially impacted by the program (positive or negatively); and to quantify the inequalities to be solved and the possible equity-focused or equity-sensitive recommendations (preferably based on evidence) to be implemented with the program. During this phase, the aim should be to diminish current inequalities, or at least, not to increase them. The researcher of the Bolivian proposal planned the program and following consideration of the most disadvantaged among the disadvantaged, proceeded to involve new players that could facilitate the implementation of the program, including children living in remote rural areas.
- 3rd Step: During the EquIR design phase, the following is suggested: to identify key players for implementing equity-focused recommendations (e.g., health professionals, patients, community, stakeholders and others); and to identify barriers and facilitators for the implementation of equity-focused recommendations. During this phase, it is relevant to consider equity-focused implementation outcomes in order to identify the best research design to evaluate the impact of implementing the program based on those outcomes (Fig. 1). In the Bolivian case, researchers will use a qualitative design to evaluate the variables that affect adherence to the Program, including families that attend the primary healthcare centers and families living in remote rural areas, visited by providers of the “Mi Salud” program. This was included in the project during the design. Researchers also included anemia and child nutrition as variables routinely monitored under the program. Identification of barriers and facilitators during this phase is the essential previous step to build on.
- 4th Step: The following is suggested for the EquIR implementation: to design strategies aimed at overcoming the barriers identified; to define resources and incentives; to define the monitoring and evaluation strategies; and to design the equity-focused communication strategies to be used in the next phase. In the Bolivian case, the researchers talked with policy-makers and government agencies in order to facilitate the participation of visitors from the “Mi Salud” program within the implementation phase of this program.
- 5th Step. In the EquIR implementation outcomes phase, it is expected that the impact of the Program will be monitored using classical implementation outcomes defined in Implementation Research [22], but these should have an equity focus, as suggested in Table 3. During this phase, it is essential to evaluate and monitor the outcomes established. In our examples, in Brazil, Colombia, Peru and the Dominican Republic, which did not explicitly include a disadvantaged population, to the use of these equity focused implementation outcomes was suggested. In the Bolivian case, they were included from the EquIR planning phase.
- 6th Step and 1st Step: The equity-focused health population status is included as the final step and the new starting point of this or any other program designed to improve inequalities. The new heath population status is the best possible outcome to monitor the implementation of health interventions or programs. However, these are long-term outcomes that are not preferred by politicians or policy-makers, or by researchers that need to know if it is convenient to continue with the program when health outcomes have not changed. In this case, the EquIR implementation outcomes are the best way to know if the program is improving health inequalities across the implementation outcomes in the short-term. If a program is not able to improve EquIR implementation outcomes in the short-term, inequalities in the health population status will not be improved in the long-term. From this perspective, the equity-sensitive IR outcomes approach would lead equity-sensitive improvements in program and policy processes that finally drive to positive population health outcomes. The iterative process proposed here with this model allows the evaluation of the impact of the program with a before-after design, emphasizing the impact on a disadvantaged population. Each of the implementation outcomes, or a set of them, could require a different kind of research design, depending on the research questions and the disadvantaged population defined from the start, during the planning phase of the program (Fig. 1).

**Table 3:** Definition of equity-focused implementation outcomes

Implementation outcomes	Equity-focused Definition
Acceptability	The perception among the key players in implementation: health professionals, stakeholders, patients, community, disadvantaged population and others.
Adoption	The intention, utilization or action to try to employ the sensitive equity recommendation in the new program or intervention.
Appropriateness	The relevance or perceived fit, or usefulness or practicability of the program or intervention in the disadvantaged population.
Feasibility	The extent to which the program or intervention allows to reduce the barriers, and can be carried out in any setting, especially among disadvantaged populations.
Fidelity	The adherence of disadvantaged population to the equity-focused implementation program or intervention.
Implementation cost	Total cost of the program implementation in disadvantaged and non-disadvantaged populations, and the final adjusted cost-effectiveness economic evaluation.
Coverage	The degree of reach, access, service spread or effective coverage (combining coverage and fidelity) on the disadvantaged population eligible to benefit from the program or the intervention.
Sustainability	The maintenance, continuation or durability of the program or intervention implemented through short, medium and long-term strategies, including disadvantaged populations.

Source: Authors, adapted from Peters DH, Adam T, Alonge O, Agyepong IA, Tran N. Implementation research: what it is and how to do it. BMJ. 2013;347:f6753 [14]

## Discussion

Although some health programs include equity issues, they do not include an implementation approach to diminish inequalities. This conceptual framework is a pragmatical proposal to incorporate equity issues during the whole process of planning, designing, implementing and monitoring the health program or intervention. This framework is based on the available evidence (Phase 1) where the relevant components and processes were identified, such as methodological steps and program planning [20, 28–30], identification of vulnerable groups [30, 34], the identification of barriers and facilitators [21, 33, 34], the design of implementation programs and tools [30, 32, 33] and monitoring and evaluation of implementation [27]. This allowed the construction of this framework which can be used as a tool to integrate equity considerations in implementation research, and making sure that equity is considered an essential outcome in health interventions, programs and policies [39]. Equity in health is an issue usually related with the development of health systems and their performance [40, 41].

We found in the literature a few frameworks that consider equity in research and reporting. One example is the PROGRESS Plus framework, which focuses on highlighting unfair differences in disease burden and interventions in order to reduce these differential effects, but which does not explicitly involve implementation considerations [37, 42]. A proposal has been made and implemented with the Development of Equity focused Clinical Practice Guidelines under the GRADE approach [43–47], although it does not relate specifically to implementation research for health programs or interventions.

Implementation Research looks for scientific evidence of programs, interventions or policies on implementation outcomes [14]. These implementation outcomes are related more to the effect of the strategies used for implementing the program, than to the effect of the program on population health status. As such, it might be more challenging to embed equity issues in implementation research proposals. Morgan et al., developed a decision-making framework to inform coverage decisions for healthcare interventions [48], involving a proposal of equal distribution of the intervention in the target region or population, using only two implementation outcomes (acceptability and feasibility). EquIR proposes equity-focused implementation outcomes, designed to measure the outcome differentially between advantaged and disadvantaged populations. Acceptability, adoption, appropriateness, feasibility, fidelity, costs, coverage or sustainability could be different for a disadvantaged population compared with an advantaged population.

Implementing new health interventions could increase health inequalities [49], and the role of EquIR is to diminish current health inequalities, not to increase the current ones, or at least, to diminish the potential negative impact on health inequalities when new interventions are implemented. It is not possible to punish new technologies because they will increase inequalities. However, implementation research could diminish this negative impact at the beginning of the implementation.

Innov8 is an approach developed to help operationalize the Sustainable Development Goals. It aims to move progressively towards universal health coverage, using evidence-based programmatic actions that help reduce in-country inequities [50] through 8 steps:

“*1. Complete the diagnostic checklist; 2. Understand the program theory; 3. Identify who is being left out by the program; 4. Identify the barriers and facilitating factors experienced by subpopulations. 5: Identify the mechanisms that give rise to health inequities; 6. Consider inter-sectorial action and social participation as central elements; 7. Produce a redesign proposal to act on the review findings; and 8. Strengthen monitoring and evaluation*” [50].

Innov8 is proposed for use with current programs in order to design or re-design them in an attempt at addressing health inequalities. Although monitoring and evaluation strategies are included to propose new changes, there are no explicit components related to implementation research or equity-focused implementation research.

EquIR offers a step-by-step proposal to facilitate the process of embedding equity issues in the implementation research of interventions or programs contained in health policies. Our experience with research projects of the country-focused program to facilitate improvements in program, policy and system implementation through research embedded within existing processes, have shown us how easy it is to forget disadvantaged populations when there is pressure to show results soon. Policy-makers are deciding what, why and with whom to implement, and “preferably sooner than later.” This proposal of a conceptual framework could facilitate the process of not forgetting disadvantaged populations when decision makers or implementers are thinking about “who to implement for” soon. Also, EquIR is an iterative process, where once the new health population status and implementation outcomes are found, it is then possible to redesign the program to enhance or strengthen the results found previously.

Health systems research usually utilizes the perspective of the institution, health professionals or third-party payers. Health equity requires that we think from different perspectives that go beyond the third payer or the health system, and in which society as a whole is involved. This is because social injustices or global inequalities are at the source of health inequities/inequalities. The role of social determinants of health is fundamental when it comes to conceiving a proposal that tries to incorporate elements aimed at minimizing inequalities or inequities in health, not just as a part of the diagnosis, but as active components that could improve the current inequalities/inequities, with the participation of other players or sectors. Education, occupation, gender, poverty, race/ethnicity, and socioeconomic condition are usually mentioned when population health status is evaluated. These relationships are well-known. However, the question is what to do. “Health in all policies” [35] is an interesting way to integrate other sectors with the health sector with a view of strengthening the role of social determinants of health in the daily practice of public health and public policies. The Innov8 approach also includes, perspectives that go beyond the health sector, with the intention of working on social determinants of health with inter-sectorial strategies [50].

## Conclusion

EquIR is a conceptual framework which is proposed for use by decision makers and researchers during the implementation of health programs, politics or interventions. EquIR involves the role of social determinants of health and the use of inter-sectorial strategies from the design of the program, which force the implementer to involve other sectors which may improve the implementation of the strategy and create a more profound impact on the equity-focused implementation outcomes and, ultimately, on inequities in the population health status, considering the close relationship among equity, social justice and social determinants of health. Future evaluation of its effectiveness to improve implementation outcomes within disadvantaged populations or, even better, to improve health outcomes in the disadvantaged population are needed.

## Data Availability

Not Applicable.

## Abbreviations

AHPSR: Alliance for Health Policy and Systems Research
CFIR: Consolidated Framework for Implementation Research
EquIR: Equity-based framework for Implementation Research
GRADE: Grading of Recommendations Assessment, Development and Evaluation
IR: Implementation Research
PAHO: Pan-American Health Organization
PARISH: Promoting Action on Research Implementation in Health Services
PROGRESS: Place of residence, Race/ethnicity/culture/language, Occupation, Gender/sex, Religion, Education, Socioeconomic status and Social capital
RAPS: Psychosocial Attention Network Qualification Program
WHO: World Health Organization

## References

[1] Nilsen P. Making sense of implementation theories, models and frameworks. Implement Sci. 2015;10:53 Available from: 10.1186/s13012-015-0242-0; https://implementationscience.biomedcentral.com/articles/10.1186/s13012-015-0242-0. 10.1186/s13012-015-0242-0 PMC4406164 25895742

[2] Vogel JP, Moore JE, Timmings C, Khan S, Khan DN, Defar A (2016). Barriers, facilitators and priorities for implementation of WHO maternal and perinatal health guidelines in four lower-income countries: a great network research activity. PLoS One.

[3] Bauer MS, Damschroder L, Hagedorn H, Smith J, Kilbourne AM (2015). An introduction to implementation science for the non-specialist. BMC Psychol.

[4] Kirchner JE, Ritchie MJ, Pitcock JA, Parker LE, Curran GM, Fortney JC (2014). Outcomes of a Partnered Facilitation Strategy to Implement Primary Care–Mental Health. J Gen Intern Med.

[5] Khan F, Owolabi M, Amatya B, Hamzat T, Ogunniyi A, Oshinowo H (2018). Challenges and barriers for implementation of the World Health Organization global disability Action plan in low- and middle- income countries. J Rehabil Med.

[6] Wensing M (2015). Implementation science in healthcare: Introduction and perspective. Z Evid Fortbild Qual Gesundhwes.

[7] Handley MA, Gorukanti A, Cattamanchi A (2016). Strategies for implementing implementation science: a methodological overview. Emerg Med J.

[8] Waters E., Hall B. J., Armstrong R., Doyle J., Pettman T. L., de Silva-Sanigorski A. (2011). Essential components of public health evidence reviews: capturing intervention complexity, implementation, economics and equity. Journal of Public Health.

[9] Arcaya Mariana C., Arcaya Alyssa L., Subramanian S. V. (2015). Inequalities in health: definitions, concepts, and theories. Global Health Action.

[10] Lee JT, Huang Z, Basu S, Millett C (2015). The inverse equity hypothesis: does it apply to coverage of cancer screening in middle-income countries?. J Epidemiol Community Health.

[11] Victora CG, Joseph G, Silva ICM, Maia FS, Vaughan JP, Barros FC (2018). The inverse equity hypothesis: analyses of institutional deliveries in 286 National Surveys. Am J Public Health.

[12] Victora CG, Vaughan JP, Barros FC, Silva AC, Tomasi E (2000). Explaining trends in inequities: evidence from Brazilian child health studies. Lancet.

[13] Alliance for Health Policy and Systems Research. Implementation Research in Three Priority Areas [Internet]. World Heal. Organ. 2017 [cited 2017 Sep 9]. Available from: http://www.who.int/alliance-hpsr/projects/ir3/en/

[14] Peters DH, Adam T, Alonge O, Agyepong IA, Tran N (2013). Implementation research: what it is and how to do it. BMJ.

[15] Damschroder LJ, Aron DC, Keith RE, Kirsh SR, Alexander JA, Lowery JC. Fostering implementation of health services research findings into practice: a consolidated framework for advancing implementation science. Implement Sci. 2009;4:50 Available from: https://implementationscience.biomedcentral.com/articles/10.1186/1748-5908-4-50. 10.1186/1748-5908-4-50 PMC2736161 19664226

[16] Curran Geoffrey M., Bauer Mark, Mittman Brian, Pyne Jeffrey M., Stetler Cheryl (2012). Effectiveness-implementation Hybrid Designs. Medical Care.

[17] Weiss CH (2017). Why do we fail to deliver evidence-based practice in critical care medicine?. Curr Opin Crit Care.

[18] Chopra M, Sharkey A, Dalmiya N, Anthony D, Binkin N (2012). Strategies to improve health coverage and narrow the equity gap in child survival, health, and nutrition. Lancet.

[19] Meyers DC, Durlak JA, Wandersman A (2012). The quality implementation Framework: a synthesis of critical steps in the implementation process. Am J Community Psychol.

[20] Kitson AL, Rycroft-Malone J, Harvey G, McCormack B, Seers K, Titchen A (2008). Evaluating the successful implementation of evidence into practice using the PARiHS framework: theoretical and practical challenges. Implement Sci.

[21] Chin MH, Goddu AP, Ferguson MJ, Peek ME (2014). Expanding and sustaining integrated health care-community efforts to reduce diabetes disparities. Heal Promot Pr.

[22] Peters DH, Tran NT, Adam T. Implementation Research in Health: A Practical Guide: WHO; 2013. p. 69. Available from: http://who.int/alliance-hpsr/alliancehpsr_irpguide.pdf. Accessed 15 June 2017.

[23] Braveman PA (2003). Monitoring equity in health and healthcare: a conceptual Framework. J Heal Popul Nutr.

[24] Tran N, Langlois EV, Reveiz L, Varallyay I, Elias V, Mancuso A (2017). Embedding research to improve program implementation in Latin America and the Caribbean. Rev Panam Salud Publica.

[25] Whitehead Margaret (1992). The Concepts and Principles of Equity and Health. International Journal of Health Services.

[26] Eslava-Schmalbach J, Elias V, Reveiz L (2017). G-ON. Incorporating health equity into implementation research: review of conceptual models. Rev Panam Salud Publica.

[27] Panamerican Health Organization/World Health Organization. Manual para el Monitoreo de las Desigualdades en Salud, con especial énfasis en países de ingresos medianos y bajos. Washington, D.C.: Panamerican Health Organization/World Health Organization. 2016. Available from: https://www.paho.org/hq/dmdocuments/2016/manual-moni-desig-sociales-salud-2016.pdf.

[28] Mann G, Squire SB, Bissell K, Eliseev P, Du Toit E, Hesseling A (2010). Beyond accuracy: creating a comprehensive evidence base for TB diagnostic tools [State of the art]. Int J Tuberc Lung Dis.

[29] Mann GH, Thomson R, Jin C, Phiri M, Vater MC, Sinanovic E (2011). The role of health economics research in implementation research for health systems strengthening. Int J Tuberc Lung Dis.

[30] Rudan I (2016). Setting health research priorities using the CHNRI method: IV. Key conceptual advances. J Glob Heal.

[31] Chin MH, Walters AE, Cook SC, Huang ES (2007). Interventions to reduce racial and ethnic disparities in health care. Med Care Res Rev.

[32] Percival NA, McCalman J, Armit C, O’Donoghue L, Bainbridge R, Rowley K, et al. Implementing health promotion tools in Australian indigenous primary health care. Heal Promot Int; 2016. Available from: http://heapro.oxfordjournals.org/content/early/2016/07/25/heapro.daw049. Accessed 10 July 2017. 10.1093/heapro/daw049 27476870

[33] Zamora G, Flores-Urrutia MC, Mayen AL, Mayén AL. Large-scale fortification of condiments and seasonings as a public health strategy: equity considerations for implementation. Ann N Y Acad Sci; 2016. Available from: https://nyaspubs.onlinelibrary.wiley.com/doi/full/10.1111/nyas.13183. Accessed 17 July 2017. 10.1111/nyas.13183 27525672

[34] Bowen S, Botting I, Roy J. Promoting action on equity issues: a knowledgeto-action handbook. Edmonton: School of Public Health, University of Alberta; 2011. Available from: http://nccdh.ca/resources/entry/promoting-action-on-equity-issues.

[35] Rudolph L, Caplan J, Ben-Moshe K, Dillon L, Framework H, Action C, et al. Health in All Policies: A guide for state and local governments [Internet]. Washington, DC and Oakland: CA Am. Public Heal. Assoc. Public Heal. Inst; 2013. Available from: https://www.apha.org/~/media/files/pdf/factsheets/health_inall_policies_guide_169pages.ashx.

[36] Marmot M (2005). Social determinants of health inequalities. Lancet.

[37] O’Neill J, Tabish H, Welch V, Petticrew M, Pottie K, Clarke M (2014). Applying an equity lens to interventions: using PROGRESS ensures consideration of socially stratifying factors to illuminate inequities in health. J Clin Epidemiol.

[38] Oliver S, Kavanagh J, Caird J, Lorenc T, Oliver K, Harden A, et al. Health promotion, inequalities and young people’s health: a systematic review of research. EPPI-Centre; 2008 [cited 2018 Jul 12]; Available from: http://researchonline.lshtm.ac.uk/2603/#.W0afkAkyAVw.mendeley

[39] Embrett MG, Randall GE (2014). Social determinants of health and health equity policy research: exploring the use, misuse, and nonuse of policy analysis theory. Soc Sci Med.

[40] Kruk ME, Freedman LP (2008). Assessing health system performance in developing countries: A review of the literature. Health Policy (New York).

[41] Senkubuge F, Modisenyane M, Bishaw T (2014). Strengthening health systems by health sector reforms. Glob Health Action.

[42] Bosch-Capblanch X, Zuske MK, Auer C (2017). Research on subgroups is not research on equity attributes: Evidence from an overview of systematic reviews on vaccination. Int J Equity Heal.

[43] Eslava-Schmalbach JH, Welch VA, Tugwell P, Amaya AC, Gaitan H, Mosquera P (2016). Incorporating equity issues into the development of Colombian clinical practice guidelines: suggestions for the GRADE approach. Rev Salud Publica.

[44] Welch VA, Akl EA, Guyatt G, Pottie K, Eslava-Schmalbach J, Ansari MT (2017). GRADE equity guidelines 1: considering health equity in GRADE guideline development: introduction and rationale. J Clin Epidemiol.

[45] Akl EA, Welch V, Pottie K, Eslava-Schmalbach J, Darzi A, Sola I, et al. GRADE equity guidelines 2: considering health equity in GRADE guideline development: equity extension of the guideline development checklist. J Clin Epidemiol; 2017. Available from: http://linkinghub.elsevier.com/retrieve/pii/S0895435617304705. Accessed 17 July 2017. 10.1016/j.jclinepi.2017.01.017 PMC6538524 28499847

[46] Welch VA, Akl EA, Pottie K, Ansari MT, Briel M, Christensen R (2017). GRADE equity guidelines 3: considering health equity in GRADE guideline development: rating the certainty of synthesized evidence. J Clin Epidemiol.

[47] Pottie K, Welch V, Morton R, Akl EA, Eslava-Schmalbach JH, Katikireddi V (2017). GRADE equity guidelines 4: considering health equity in GRADE guideline development: evidence to decision process. J Clin Epidemiol.

[48] Morgan RL, Kelley L, Guyatt GH, Johnson A, Lavis JN (2018). Decision-making frameworks and considerations for informing coverage decisions for healthcare interventions: a critical interpretive synthesis. J Clin Epidemiol.

[49] Guthmann J-P, Pelat C, Célant N, Parent du Chatelet I, Duport N, Rochereau T (2017). Socioeconomic inequalities to accessing vaccination against human papillomavirus in France: Results of the Health, Health Care and Insurance Survey, 2012. Rev Epidemiol Sante Publique.

[50] World Health Organization. Innov8 approach for reviewing national health programmes to leave no one behind: technical handbook [Internet]. 2016. Available from: http://www.who.int/life-course/partners/innov8/innov8-technical-handbook/en/. Accessed 17 July 2017.

